# Reproductive outcomes after expectant management of enhanced myometrial vascularity secondary to first trimester retained products of conception: A follow‐up study

**DOI:** 10.1002/ijgo.70213

**Published:** 2025-05-12

**Authors:** Anjeza Xholli, Umberto Scovazzi, Ambrogio P. Londero, Filippo Molinari, Isabella Perugi, Alessandra De Ponti, Angelo Cagnacci

**Affiliations:** ^1^ Academic Unit of Obstetrics and Gynecology IRCCS Hospital Polyclinic San Martino Genoa Italy; ^2^ Department of Neurology, Rehabilitation, Ophthalmology, Genetics Maternal and Infant Health (DiNOGMI) Genoa Italy; ^3^ Obstetrics and Gynecology Unit IRCCS Istituto Giannina Gaslini Genoa Italy

**Keywords:** abnormal uterine bleeding, abortion, arterio‐venous malformation, enhanced myometrial vascularity, metrorrhagia, miscarriage, retained products of conception, termination of pregnancy

It has been reported that after a termination of pregnancy (TOP) or the management of a non‐viable pregnancy or miscarriage the presence of retained products of conceptions (RPOC) and their removal does not affect future reproductive and obstetrical outcomes.^1^, ^2^ Enhanced myometrial vascularity (EMV) following first trimester TOP or miscarriage is frequently associated with visible RPOC and it can be managed either expectantly,^3^ or surgically (Figure S1).^4^, ^5^ Following the development of new technologies and a higher sensitivity of ultrasound, the prevalence of EMV following TOP is higher than previously expected (up to 35%) and in most cases spontaneously resolve.^3^ Whether the development of EMV and its spontaneous resolution influence successive reproductive and obstetrical outcomes is presently unknown and it was herein evaluated.

In two previous studies^3^, ^6^ performed in our institution in the period 2021–2023, women developing EMV after first trimester TOP or miscarriage were prospectively evaluated until spontaneous resolution. The investigations that allowed long‐term follow‐up evaluations were approved by the local ethics committee (CER Liguria 304/2022, DB id 12 400), and all included women had signed a consent form for the use of their anonymous data. Between April and June 2024, investigators of the original research (US, IP, ADP) contacted those women that had developed EMV and were previously followed until spontaneous resolution, asking on the aftermath research of a pregnancy, achievement of a pregnancy, eventual obstetric outcomes and live births. Pregnancy rate (PR) was calculated as the total number of women who conceived among women trying to get pregnant. Live birth rate (LBR) was calculated as the capability among women who conceived to deliver (naturally or surgically) at least one live newborn (i.e., women with an alive newborn out of women who conceived). Secondary outcomes of the study were the analysis of modifications of the menstrual cycle after EMV resolution, eventual subsequent TOP, time elapsed between EMV resolution and first eventual conception, any obstetrical condition/complication during pregnancy following an EMV resolution, type of delivery (vaginal or cesarean) and gestational age at delivery.

Analyses were performed using the statistical software package StatView 5.01 (SAS Institute Inc., Cary, NC, USA); a *P* value of less than 0.05 was considered statistically significant.

Overall, 68 patients with previous EMV were included. A total of 55 patients developed an EMV after a TOP and 13 after the management of a non‐viable pregnancy or miscarriage. The patient characteristics are reported in Table 1. Among these, 62 women were successfully contacted, of whom 40 reported they did not try to conceive. Among the remaining 22 (35.5%), 16 (73%) conceived, and six did not. Among these, four were still trying but for less than one year, and one had a history of infertility. One of the 16 women had previously (but after EMV) terminated a pregnancy at 20 weeks of gestation for fetal malformation, and another had conceived a second time during the follow‐up period. Finally, of the 16 women who conceived, three women had a miscarriage (18%), and 13 women (81.3%) successfully delivered at term, one by repeated cesarean delivery. One woman developed mild gestational diabetes and two developed a PROM. Postpartum hemorrhage (PPH) occurred in two (15.4%) women and was resolved by curettage in one case and by the manual removal of the placenta in the other (Table 1).

**TABLE 1:** Characteristics of patients in the post hoc analysis and comparison between women who did and did not conceive.

Characteristics of 62 patients reached for interview (mean ± SD)			
Age (years)		31.7 ± 6.1	
Weight (kg)		60.8 ± 9.6	
Height (cm)		162.4 ± 6.2	
Time between TOP/management of non‐viable pregnancy/miscarriage to EMV diagnosis (weeks)		5.8 ± 2.9	
TOP		49 (79%)	
Management of non‐viable pregnancy and miscarriage		13 (21%)	
Expectant management of EMV		57 (91.9%)	
Dilation and curettage of EMV		5 (8.1%)	
EMV volume (cm^3^)		2.9 ± 3.2	
EMV PSV (cm/s)		28.8 ± 5.1	
Nulliparous		21 (33.9%)	
Menstrual changes for at least 6 months following resolution of EMV		21/51 (41.2%)^a^	
Women wishing to conceive		22 (35.5%)	
PR		16/22 (73%)^b^	
LBR		13/16 (81.3%)^c^	
Time elapsed between EMV resolution and LMP of pregnancy (months)		12.1 ± 8.3	
Spontaneous delivery		11 (91.7%)	
Cesarean section		1 (8.3%)	
Pathology in pregnancy (among LBR+)		3/13 (23.1%)	
Complications after delivery		2/13 (15.4%)	
PPH + curettage		1/13 (7.7%)	
PPH + manual removal of placenta		1/13 (7.7%)	
Comparison between women who conceived (*n* = 16) and who did not (*n* = 6)			
	Yes	No	*P* value
Age (years)	33.8 ± 7.5	32.4 ± 4.8	0.775
Weight (kg)	63.0 ± 10.0	61.7 ± 8.2	0.757
Height (cm)	162.3 ± 6.9	165.0 ± 6.0	0.370
Time between TOP/management of non‐viable pregnancy/miscarriage to EMV diagnosis (weeks)	5.0 ± 0.63	5.61 ± 1.53	0.360
EMV volume (cm^3^)	3.7 ± 4.7	3.2 ± 3.1	0.740
EMV PSV (cm/s)	27.0 ± 3.4	30.0 ± 6.0	0.269

Abbreviations: EMV, enhanced myometrial vascularity; LBR, live birth rate; PPH, postpartum hemorrhage; PR, pregnancy rate; PSV, peak systolic velocity; SD, standard deviation; TOP, termination of pregnancy.

^a^ Any menstrual change including hypermenorrhea, ipermenorrhea and oligomenorrhea, among women not taking hormonal therapy.

^b^ PR was calculated as the total number of pregnancies achieved among women wishing to conceive.

^c^ The LBR was calculated as the total number of live births per number of women achieving pregnancy.

Our post hoc analysis indicates a high PR after the spontaneous resolution of EMV (i.e., 73%) and high LBR (81.3%). The PR and LBR of our cohort were as high as those of healthy women 6 to 24 months after a miscarriage.^7^ The overall obstetrics and placental complications were similar to those reported after the surgical removal of RPOC.^1^ In this unique, albeit not huge, cohort of women, the spontaneous resolution of EMV following first trimester TOP or miscarriage did not seem to affect successive reproductive and obstetrical outcomes.

## AUTHOR CONTRIBUTIONS

A.X., U.S. and A.C. design of the study, data curation, execution, analysis, manuscript drafting, critical discussion, and final approval of the manuscript. F.M., I.P. and A.P.L. execution, analysis, and final approval of the manuscript. A.D.P. data curation and final approval of the manuscript.

## FUNDING INFORMATION

No specific grant was given to support the current study.

## CONFLICT OF INTEREST STATEMENT

The authors report no conflict of interest.

## Supporting information


**Figure S1.** Representation of enhanced myometrial vascularity – Figure S1: Overview of enhanced myometrial vascularity composition within a thickened, non‐homogeneous, endometrial cavity, with high vascularization shown at the Power Doppler assessment (a); the respective microstructural area is composed mainly by mixed vessels and thrombosed vessels, with the presence also of chorionic villi (white circle) – image with magnification 2.5× (b).

## Data Availability

The data that support the findings of this study are available from the corresponding author upon reasonable request.
