# Supplementary figures and images for: Reproductive outcomes after expectant management of enhanced myometrial vascularity secondary to first trimester retained products of conception: A follow‐up study
Source: Int J Gynaecol Obstet. 2025 May 12;171(1):468–70. doi: 10.1002/ijgo.70213 (PMC12447666; doi:10.1002/ijgo.70213)

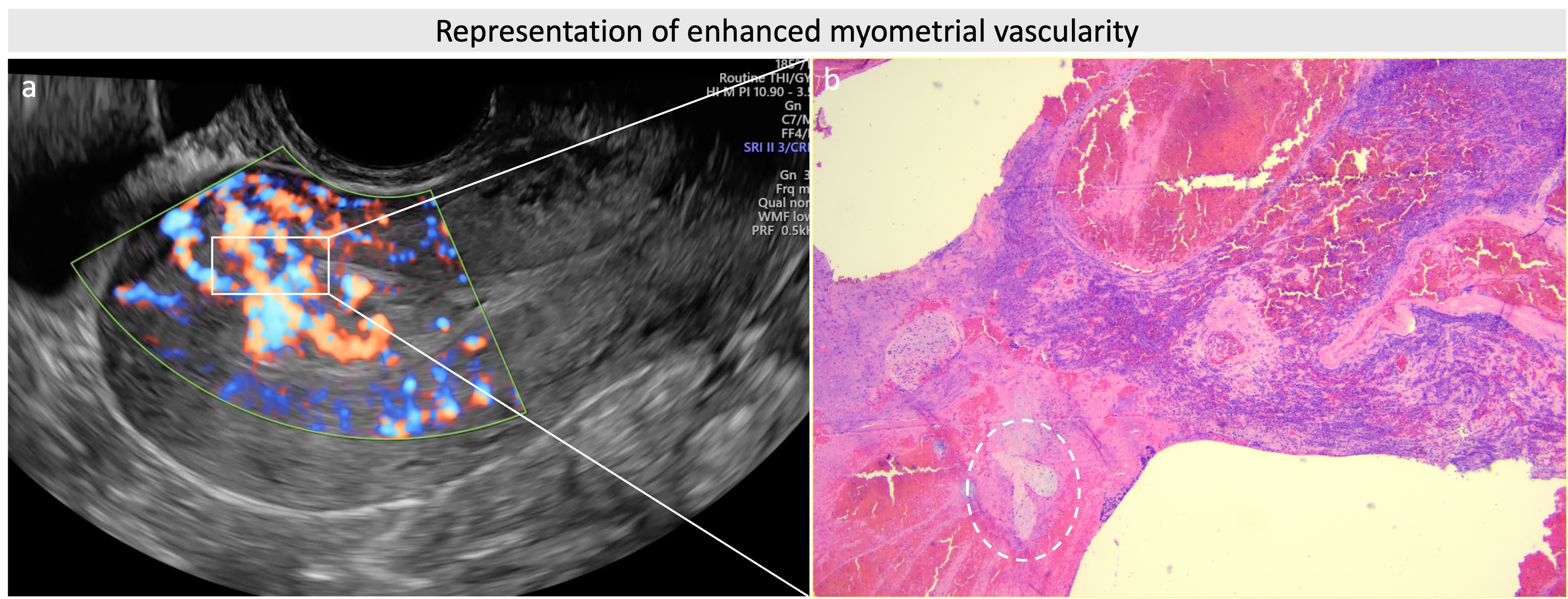

Supplement: Supplementary file 1 — Figure S1. Representation of enhanced myometrial vascularity – Figure S1: Overview of enhanced myometrial vascularity composition within a thickened, non‐homogeneous, endometrial cavity, with high vascularization shown at the Power Doppler assessment (a); the respective microstructural area is composed mainly by mixed vessels and thrombosed vessels, with the presence also of chorionic villi (white circle) – image with magnification 2.5× (b). [file IJGO-171-468-s001.jpg]
